# Evaluation and Prediction of Low-Carbon Economic Efficiency in China, Japan and South Korea: Based on DEA and Machine Learning

**DOI:** 10.3390/ijerph191912709

**Published:** 2022-10-04

**Authors:** Huayong Niu, Zhishuo Zhang, Manting Luo

**Affiliations:** International Business School, Beijing Foreign Studies University, Beijing 100089, China

**Keywords:** low-carbon economy, low-carbon economic efficiency, data envelopment analysis, machine learning

## Abstract

Addressing global climate change has become a broad consensus in the international community. Low-carbon economic development, as an effective means to address global climate change issues, has been widely explored and practiced by countries around the world. As major carbon emitting countries, there has been much focus on China, Japan and South Korea, and it is of practical significance to study their low-carbon economic development. To further measure their trend of low-carbon economic development, this paper firstly constructs a low-carbon economic efficiency evaluation index system and uses the Slack Based Measure (SBM) model. This is a kind of data envelopment analysis (DEA) method, with undesirable output based on global covariance to measure the low-carbon economic efficiency of 94 provincial-level administrative divisions (PLADs) in China, Japan, and South Korea from 2013 to 2019. Subsequently, this paper uses 10 mainstream machine learning models and combining them with Grid Search with Cross Validation (GridSearchCV) methods, selects the machine learning model with the best prediction effect. The model predicts the low-carbon economic efficiency of PLADs in China, Japan, and South Korea from 2020 to 2024 based on the parameter configuration for the best prediction effect. Finally, according to the research results, this paper proposes targeted advice for regionalized cooperation on low-carbon economic development in China, Japan, and South Korea to jointly address global climate change issues.

## 1. Introduction

Global climate change is one of the most serious challenges to modern human survival and development. It not only brings about natural problems such as rising temperatures and melting glaciers, but also triggers global issues such as food security, energy security and ecological security. Carbon dioxide, as the main greenhouse gas, is the most important cause of global climate change. In the process of coping with global climate change, countries are striving to explore effective paths for synergistic development of economic growth and low carbon. Since the concept of “low carbon economy” was first proposed in the British Energy White Paper “Our Energy Future: Creating a Low Carbon Economy” [1] in 2003, this economic model has been advocated by various countries. A low carbon economy refers to a more efficient use of resources to improve the standard of living and quality of life. It advocates a sustainable economic development model based on low energy consumption, low pollution and low emissions. It can be seen that the low carbon economy is an important way to guarantee people’s quality of life and achieve sustainable economic development, and it is also an inevitable choice to mitigate the negative effects brought by global climate change. The low-carbon economic efficiency studied in this paper examines the coordinated relationship between capital, labor and energy inputs and economic outputs under the CO_2_ emission constraint, which is an important way to measure the results of low-carbon economic development in each region.

The Asia-Pacific region is currently the region with the highest CO_2_ emission, among which China is currently the largest CO_2_ emitter in the world, and Japan and South Korea are also among the top CO_2_ emitters [2]. As the core force of cooperation in the Asia-Pacific region, China, Japan, and South Korea have strong complementarities in resources, markets, and technologies to jointly address global climate change. As an effective unit of global cooperation and a useful supplement to promote international cooperation, regional cooperation between China, Japan and South Korea will also have a significant impact on addressing global climate change issues. This paper examines the low-carbon economic efficiency of provincial-level administrative divisions in China, Japan and South Korea, with the aim of exploring the low-carbon economic development in each region and proposing a targeted cooperation program for regional low-carbon economic development.

This paper selects labor force, capital stock, total energy consumption, regional GDP and carbon dioxide emissions as input-output indicators, thus constructing a low-carbon economic efficiency evaluation index system. The research uses a Slack Based Measure (SBM) model with undesirable output based on global covariance to measure the low-carbon economic efficiency values of 94 provincial-level administrative divisions (PLADs) in China, Japan, and South Korea from 2013 to 2019. The efficiency value series from 2013–2017 and 2014–2018 are used as the input of the training samples, and the efficiency values of 2018 and 2019 are used as the output of the training samples, resulting in a total of 188 training samples. Based on the training samples, this paper uses 10 mainstream machine learning models combined with the Grid Search with Cross Validation (GridSearchCV) method to find the model with the best prediction effect. Based on the model and parameter configuration under the best prediction effect, this paper predicts the low-carbon economic efficiency values of PLADs in China, Japan, and South Korea for the period of 2020–2024. Finally, on the basis of the results, this paper proposes targeted recommendations to promote low-carbon economic development in China, Japan, and South Korea.

The main contributions of this paper are: (1) The current studies of cross-country low-carbon economic efficiency mainly use national-level data, and researches on PLADs are mainly focused on China. There is a lack of cross-country research focusing on PLADs, and this research will be further expanded in this paper. (2) This paper adopts the SBM model with undesirable output based on global covariance, which can make the efficiency comparison of decision-making units in all years based on the same effective frontier, and achieve the comparability of decision-making units among different years. (3) The data envelopment analysis (DEA) method is based on past input-output indicators to evaluate the current low-carbon economic efficiency, and it is difficult to use this method to predict future efficiency. This paper uses 10 machine learning models combined with GridSearchCV method to compare predictions for the PLADs in China, Japan and South Korea from 2020 to 2024. The prediction is based on the model and parameter configuration under the best prediction effect, which makes up for the shortcomings of DEA method and provides a reference for scientific decision-making. (4) This study is a methodological expansion in the field of efficiency evaluation and prediction, combining DEA methods and machine learning methods to form an efficiency prediction model with good fitting ability and generalization ability.

This paper is structured as follows: The second part discusses relevant literature in this field. The third part introduces the basic models and methods involved in this study. The fourth part designs the evaluation index system and performs pre-processing of the index data. The fifth part analyzes the evaluation and prediction results of low-carbon economic efficiency. The sixth part summarizes the main conclusions and makes corresponding suggestions based on the findings. Finally, this paper points out the limitations of the current study and provides a perspective on future research directions.

## 2. Literature Review

### 2.1. Economic Efficiency Evaluation Studies

#### 2.1.1. Low-Carbon Economic Efficiency Evaluation Indicators

This paper calculates low-carbon economic efficiency through input and output indicators, so the settings of input and output indicators are very important for the evaluation. This paper summarizes the existing relevant research literature, and Table 1 outlines the input and output indicators in this literature, which will provide the basis for the construction of the low-carbon economic efficiency evaluation index system in this paper.

**Table 1:** Input and output indicators in some papers on low-carbon economic efficiency.

Authors/Year	Inputs	Outputs
Hu and Kao (2007) [3]	Labor input, capital input, energy input	Gross domestic product
Zhou and Ang (2008) [4]	Capital stock, labor force	Gross domestic product, CO_2_ emissions
Wang et al. (2012) [5]	Capital input, labor input, energy input	The gross product value of industrial enterprises above a designated size
Wang et al. (2013) [6]	Energy consumption, labor input, capital input	Gross domestic product, CO_2_ emissions
Wang and Wei (2014) [7]	Labor input, capital input, energy input	Total volume of industrial sulfur dioxide emissions, total volume of industrial carbon dioxide emissions
Wang et al. (2014) [8]	Capital stock, energy consumption, labor	Gross domestic product, environmental pollutants
Wang and Feng (2014) [9]	Capital stock, labor, energy consumption	Gross domestic product, positive environmental indicator
Zhang et al. (2017) [10]	Labor employment, capital stock, total energy consumption	Gross domestic product, CO_2_ emissions
Dong et al. (2017) [11]	Capital stock, labor, CO_2_ emissions	Gross domestic product
Cheng et al. (2019) [12]	Labor, capital stock, energy consumption	Gross domestic product, CO_2_ emissions
Li et al. (2020) [13]	Labor, capital stock, energy consumption	Gross domestic product, CO_2_ emissions
Wang et al. (2021) [14]	Labor, capital stock, energy consumption	Gross domestic product, CO_2_ emissions
Xue et al. (2022) [15]	Manpower input, capital investment, energy input	Gross domestic product, CO_2_ emissions
Niu et al. (2022) [16]	Labor force, capital stock, total energy consumption	Gross regional product, carbon dioxide emissions

#### 2.1.2. Low-Carbon Economy Efficiency Evaluation Methods

In terms of low carbon economic efficiency evaluation methods, the frontier analysis method is the mostly used. It mainly includes two methods, Stochastic Frontier Analysis (SFA) [17] and DEA [18]. The difference between them is that SFA method is a parametric method, which needs to set the production function form in advance. If the function form is set wrong, it will cause measurement errors. However, the DEA method is a non-parametric method, which does not need to set the function form in advance, and it is applicable to the production function model with multiple inputs and outputs. Therefore, the DEA method is the most popular method for low-carbon economic efficiency evaluation.

Xi and Li [19] measured the low-carbon economic efficiency of seven economic regions in China in 2006 and 2007, using the CCR model based on constant returns to scale in the DEA method. Fan and Fang [20] considered the undesirable output indicators, and took the reciprocal of the undesirable output indicators as the desirable output to complete the evaluation index system. Then they used the CCR model and the BCC model based on variable returns to scale to evaluate the level of circular economic development of Chinese provinces in 2017, and compared the scale efficiency of the two models. However, it should not be ignored that the CCR and BCC models are radial DEA models, which cannot measure the full range of slack variables. Liu et al. [21] measured CO_2_ emission efficiency of 30 provinces in China from 2000 to 2011 using the SBM model with undesirable output. The above models yielded an efficiency value of up to 1. If there are many decision-making units in an efficient state, these models will not be able to further distinguish them. Some studies [10,22,23] combined super efficiency and SBM models to form a Super-SBM model to distinguish decision-making units with an efficiency value of 1. The efficiency values measured by the radial DEA, the SBM and Super-SBM models are all based on cross-sectional data, and each set of cross-sectional data constitutes a different effective frontier. If intertemporal comparisons are required, the efficiency values cannot simply reflect changes in their own technical efficiency, but also include the impact of changes in the effective frontier on the efficiency values. Therefore, some scholars used the window DEA model to evaluate low-carbon economic efficiency [24,25,26], which took all the decision-making units in the window period as the reference set and constructed the effective frontier, so as to achieve the intertemporal comparability within the window period. Nevertheless, the window DEA model still does not achieve a global covariance.

In summary, this paper selects the SBM model with undesirable output based on global covariance to evaluate the low-carbon economic efficiency in China, Japan and South Korea. The model makes the efficiency measurement more accurate compared with the radial DEA model and achieves the comparability among decision-making units in different years. There are 21 PLADs achieving efficiency values of 1 in different years, which accounts for only 3.19% of the 658 evaluation results (94 decision-making units per year × 7 years = 658), so the research does not use Super-SBM model for evaluation. This also avoids its computational drawback, i.e., the problem of no feasible solution may occur.

### 2.2. Low-Carbon Economic Efficiency Predicting Studies

There are very limited studies on low-carbon economic efficiency prediction, and most studies stop at evaluation. Niu et al. [16] used a time-recursive neural network model to predict the efficiency of carbon emissions and achieved good prediction results. Some scholars have focused on predicting the input or output variables in the evaluation index system of low-carbon economic efficiency. Wang and Li [27] used a GM(1,1) gray prediction model to predict energy consumption in Hebei, China. Pao et al. [28] used an improved gray prediction model to predict carbon dioxide emissions, energy consumption, and economic growth in China. In addition to gray models, some scholars used an Autoregressive Integrated Moving Average (ARIMA) model for forecasting: Nyoni and Bonga [29] used annual time series data of CO_2_ emissions in India from 1960–2017 to predict the emissions in the period 2018–2030. Ning et al. [30] selected Beijing, Henan, Guangdong, and Zhejiang in China, and used ARIMA model to forecast CO_2_ emissions and trends in the next three years based on CO_2_ emissions data from 1997–2017. Lotfalipour [31] used a gray prediction model and an ARIMA model to predict CO_2_ emissions in Iran, comparing the prediction effects of two models, and found that the gray prediction model has better prediction effects. With the rise of machine learning techniques, these are widely used in the field of forecasting. Rehman et al. [32] used a neural network model for forecasting CO_2_ emissions from energy, transport and manufacturing sectors of Pakistan. Ma et al. [33] applied a support vector machine approach for forecasting building energy consumption in China and achieved good prediction results with generalization ability of the model. Bakay and Ağbulut [34] used electricity production data of Turkey to forecast greenhouse gas emissions based on deep learning, support vector machine and artificial neural network algorithms. Further, some studies have compared machine learning methods with ARIMA models [35,36,37] and also machine learning methods with gray prediction models [38,39,40], where machine learning methods possess higher prediction accuracy. Both gray prediction models and ARIMA are essentially linear regression models, whereas machine learning models can generally handle both linear and nonlinear relationships in sample data, and this feature determines the greater advantage of machine learning models in prediction.

Through the literature review, it was found that there are very limited related researches in the field of low-carbon economic efficiency prediction, but that the prediction of some input or output indicators in the low-carbon economic efficiency evaluation index system have achieved satisfactory results, which provide support for efficiency prediction. This paper further selects 10 mainstream machine learning models for prediction of low-carbon economic efficiency; the models cover traditional machine learning models, tree-based machine learning models and integrated machine learning models.

## 3. Modeling Method

### 3.1. SBM Model with Undesirable Output Based on Global Covariance

#### 3.1.1. SBM Model with Undesirable Output

In 1978, Charnes, Cooper, and Rhodes [18] proposed the first DEA model, the CCR model, which was named using the three individuals’ initials of last names. They extended the concept of single-input, single-output engineering efficiency to the evaluation of the relative efficiency of multiple inputs and multiple outputs. Since the CCR model belongs to the radial DEA model, the efficiency values of non-effective decision-making units only reflect the part of equal proportional improvement, while the part of slack improvement is not reflected in the measurement. For consideration of improvements, Tone [41] proposed the non-oriented SBM model with the planning equation of Equation (1), which measures the inefficiency condition from both input and output perspectives. This model takes into account the input-output slack problem, which makes the measurement results more accurate.

(1) $$\begin{array}{cc} \min\rho & =\frac{1-\frac{1}{m}\sum_{i=1}^{m}\frac{s_{i}^{-}}{x_{ik}}}{1+\frac{1}{q}\sum_{r=1}^{q}\frac{s_{r}^{+}}{y_{rk}^{}}}\\ s.t.\; & \sum_{j=1}^{n}x_{ij}\lambda_{j}+s_{i}^{-}=x_{ik}\\ & \sum_{j=1}^{n}y_{rj}^{g}\lambda_{j}-s_{r}^{+}=y_{rk}^{}\\ & s^{-},s^{+},\lambda \geq 0 \end{array}$$

In the formula, ρ is the objective function, that is, the efficiency value of the decision-making unit; its value range is (0, 1]. $x_{ik}(i=1,2,\ldots ,m)$ represents the *ith* input of the *kth* decision-making unit. $y_{rk}^{}(r=1,2,\ldots ,q)$ represents the *rth* output of the *kth* decision-making unit. λ represents the weight vector, and $\left(\sum_{j=1}^{n}x_{ij}\lambda_{j},\sum_{j=1}^{n}y_{rj}^{g}\lambda_{j}\right)$ is the objective value of the *kth* decision-making unit being evaluated. $s^{-}$ and $s^{+}$ is the input slack variable and output slack variable. When and only when ρ=1, $s^{-}=0$ and $s^{+}=0$, the decision-making unit is valid. Otherwise, the decision unit is relatively non-valid.

To solve the problem that the SBM model cannot measure the efficiency of the decision-making unit with undesirable outputs, Tone [42] then proposed the SBM model with undesirable outputs, and its planning equation is Equation (2). Compared to Equation (1), *q_1_* outputs are further divided into *q*_1_ desirable outputs and *q*_2_ undesirable outputs, $s^{-}$, $s^{g}$ and $s^{b}$ are input slack variables, desirable output slack variables, and undesirable output slack variables, respectively. The decision-making unit is valid when and only when ρ=1, $s^{-}=0$, $s^{g}=0$ and $s^{b}=0$.

(2) $$\begin{array}{cc} \min\rho & =\frac{1-\frac{1}{m}\sum_{i=1}^{m}\frac{s_{i}^{-}}{x_{ik}}}{1+\frac{1}{q_{1}+q_{2}}\left(\sum_{r=1}^{q_{1}}\frac{s_{r}^{g}}{y_{rk}^{g}}+\sum_{r=1}^{q_{2}}\frac{s_{r}^{b}}{y_{rk}^{b}}\right)}\\ s.t.\; & \sum_{j=1}^{n}x_{ij}\lambda_{j}+s_{i}^{-}=x_{ik}\\ & \sum_{j=1}^{n}y_{rj}^{g}\lambda_{j}-s_{r}^{g}=y_{rk}^{g}\\ & \sum_{j=1}^{n}y_{rj}^{b}\lambda_{j}+s_{r}^{b}=y_{rk}^{b}\\ & s^{-}\geq 0,s^{g}\geq 0,s^{b}\geq 0,\lambda \geq 0 \end{array}$$

#### 3.1.2. Global Covariance Method

Since the efficiency values measured by the SBM model with undesirable output are based on different effective frontiers in different years, it is not possible to compare the efficiency values in different years. This paper combines the global covariance method [43], which treats the same decision-making unit in different years as different decision-making units, so as to construct an effective frontier for efficiency measurement based on all panel data, and this method can achieve comparability of decision-making units between different years.

### 3.2. Machine Learning Models

Based on the regression problem, this paper selects 10 mainstream machine learning models with supervised learning. These are then combined with GridSearchCV method for parameter tuning and prediction. Machine learning models cover traditional machine learning models, tree-based machine learning models and integrated machine learning models, and this section briefly introduces the main contents of these models.

#### 3.2.1. Linear Regression (LR)

LR [44] is a typical regression model that predicts the target y by Equation (3), where $\hat{y}$ is the predicted value of the predicted target y, $x=\left(x_{1},\cdots x_{p}\right)$ is the input characteristic variable, $w=\left(w_{1},\cdots w_{p}\right)$ is the weight vector, and $w_{0}$ is the intercept term in the linear regression model.

(3) $$\hat{y}(w,x)=w_{0}+w_{1}x_{1}+\ldots +w_{p}x_{p}$$

#### 3.2.2. Support Vector Machine (SVM)

SVM [45] is a supervised algorithm that can be used for classification and regression problems, and it shows many unique advantages in solving small sample, nonlinear, and high-dimensional pattern recognition. The objective function of the SVM model is shown in Equation (4), where *n* represents *n* data points, w is a normalization vector, *C* represents the penalty parameter, and the kernel function f(x) is used to measure the similarity between two data points $x_{i}$ and $x_{j}$. The common types of kernel functions include: linear kernels function, radial basis function, polynomial kernels function, and sigmoid kernels function.

(4) $$\arg\underset{w}{\min}\left\{\frac{1}{n}\sum_{i=1}^{n}\max\left\{0,1-y_{i}f\left(x_{i}\right)\right\}+Cw^{T}w\right\}$$

#### 3.2.3. Back Propagation Neural Network (BPNN)

BPNN [46] is a widely used neural network model, consisting of an input layer, an implicit layer, and an output layer. Through the training of sample data, BPNN constantly corrects the network weights and thresholds, and iterates repeatedly until it reaches the minimum error sum of squares and approximates the desired output.

#### 3.2.4. Decision Tree (DT)

DT is a tree structure, and this paper adopts the CART (Classification And Regression Tree) algorithm [47] for the regression problem. CART mainly divides the feature space into a number of non-overlapping regions, and each division cell has a specific output. By assigning the sample data to a cell according to its characteristics, the corresponding prediction value is obtained, which is the arithmetic average of the values taken by each sample in the training set in that region. The measure of the division is the mean square error of the labeled and regression values of the samples, positioned in Equation (5), where *n* represents the training sample set with *n* samples, $y_{i}$ represents the labeled value of the *ith* sample, and $\bar{y}$ represents the mean value of all samples.

(5) $$E(D)=\frac{1}{n}\sum_{i=1}^{n}{\left(y_{i}-\bar{y}\right)}^{2}$$

#### 3.2.5. Random Forest (RF), Gradient Boosting Decision Tree (GBDT), Extreme Gradient Boosting (XGBoost) and Light Gradient Boosting Machine (LightGBM)

Single decision trees are often unsatisfactory for fitting complex data, while integrated learning methods based on decision trees use decision trees as individual learners. By combining multiple learners, integrated learning models based on decision trees can generally obtain better generalization performance than individual learners. Common integrated decision tree-based learning methods include RF, GBDT, XGBoost and LightGBM.

RF [48] adds the random selection of features to the random sampling of samples in Bagging. When dealing with regression problems, for one input sample, N trees will have N outputs, and the mean value of each decision tree output is the final output of the random forest.

GBDT [49] is an iterative decision tree algorithm. The core of GBDT is that in each iteration, the latter decision tree is trained using the residuals of the previous decision trees following the negative gradient. The negative gradient residuals can be calculated by Equation (6).

(6) $$r_{ti}=-{\left[\frac{\partial L\left(y,f\left(x_{i}\right)\right)}{\partial f\left(x_{i}\right)}\right]}_{f(x)=f_{t-1}(x)}$$

where $r_{ti}$ denotes the negative gradient of sample *i* at the iteration of *t*th times. $L\left(y,f\left(x_{i}\right)\right)$ represents the loss function, which can be expressed as Equation (7).

(7) L(y,f(x))=log(1+exp(−yf(x))

XGBoost [50] adds a regularization term to the objective function to avoid overfitting. The model is shown in Equation (8), where *k* denotes the number of decision trees in the model, $x_{i}$ denotes the *i^th^* input sample, ${\hat{y}}_{i}$ denotes the predicted value of the training sample after the *kth* iteration, $f_{k}\left(x_{i}\right)$ denotes the predicted value of the *kth* tree, and *F* is the set of all decision trees.

(8) $${\hat{y}}_{i}=\sum_{k=1}^{K}f_{k}\left(x_{i}\right),\;f_{k}\in F$$

LightGBM [51] takes GBDT as its core. It uses Gradient-based One-Side Sampling (GOSS) and Exclusive Feature Bundling (EFB) to adapt the algorithm to high-dimensional data and improve its computational efficiency by making some improvements on sampling methods and feature merging.

#### 3.2.6. Adaptive Boosting (AdaBoost) and Bootstrap Aggregating (Bagging)

In addition to the machine learning model based on decision trees in Section 3.2.5, there are two mainstream integrated learning models, namely AdaBoost and Bagging.

Adaboost [52] was proposed by Freund and Schapire. The base classifier of this model is weighted, and the final prediction result is a weighted sum of the base classifier prediction results. The weight values of the training samples are dynamically updated by each iteration, and the samples with large error rates will have their weight values increased in the next iteration. The model focuses on the training samples with large error rates.

Bagging [53] performs multiple sampling of the training sample set, each with a put-back. Then it uses the sample dataset formed by each sampling to train the base learner, and calculates the mean value of the prediction results of each base learner as the final prediction result. Compared with the way Adaboost decides the weight values of the training samples based on the error rate situation, Bagging considers each training sample to have equal weight values.

#### 3.2.7. GridSearchCV

To be able to fit and predict better, we need to tune the parameters of the machine learning model. GridSearchCV [54] provides us with a parameter tuning method, which consists of two parts: grid search and cross validation. Among them, grid search searches for combinations of parameter configurations, i.e., adjusting different parameters sequentially in steps within a specified parameter range, and using the adjusted parameter combinations to train the machine learning model, so as to find the parameter combination with the highest accuracy in the test set. In this paper, the coefficient of determination R^2^ is used as a measure to evaluate the accuracy of the regression model. For regression models, R^2^ is the best indicator of the predictive effect because Root Mean Squared Error (RMSE) and Mean Absolute Error (MAE) both suffer from the absence of upper and lower bounds [55]. R^2^ represents the ratio of the explained sum of squares of deviations to the total sum of squares in the model, and the formula is expressed as Equation (8). $y^{\left(i\right)}$ is the true value, ${\hat{y}}^{\left(i\right)}$ is the predicted value, $\sum_{i}{\left({\hat{y}}^{\left(i\right)}-y^{\left(i\right)}\right)}^{2}$ is the error arising from the prediction, and $\sum_{i}{\left(\bar{y}-y^{\left(i\right)}\right)}^{2}$ is the error arising from the mean. When our prediction model does not have any error, R^2^ gets the maximum value of 1. When our model is equal to the benchmark model, R^2^ = 0. When R^2^ < 0, it means that the model is not as good as the benchmark model.

(9) $$R^{2}=1-\frac{\sum_{i}{\left({\hat{y}}^{\left(i\right)}-y^{\left(i\right)}\right)}^{2}}{\sum_{i}{\left(\bar{y}-y^{\left(i\right)}\right)}^{2}}$$

In order to avoid the influence of the randomness of the training samples on the accuracy, in this paper, the R^2^ of parameter combinations in each group is evaluated by grid search combining the ten-fold cross-validation method. We divide all the data in the training set into 10 equal parts, take 1 part as the validation set, and the remaining 9 parts as the training set for cross-validation. Finally, we get 10 R^2^, and take their mean values as the final R^2^ of parameter combinations. We take the one with the highest R^2^ as the parameter setting of the subsequent prediction model.

## 4. Evaluation Index System and Research Data

### 4.1. Decision-Making Unit

This paper takes 94 PLADs in China, Japan and South Korea as decision-making units, including: (1) Provinces, municipalities, and autonomous regions in mainland China. Due to the lack of data in the Tibet Autonomous Region, there are 30 decision-making units in total; (2) Prefectures in Japan, a total of 47 decision-making units; (3) Special city, special municipality, metropolitan cities, provinces and special autonomous provinces in South Korea, a total of 17 decision-making units.

### 4.2. Low-Carbon Economic Efficiency Evaluation Index System

According to the literature review above in “2.1.1 Low-Carbon Economic Efficiency Evaluation Indicators”, this paper finally selects labor, capital stock and total energy consumption as input indicators, the desirable output indicator is GDP, and the undesirable output indicator is carbon dioxide emissions. The specific measurement methods and units of the input-output indicators are shown in Table 2.

### 4.3. Data Sources and Descriptive Statistics

The data sources of input-output indicators are shown in Table 3. The indicator data from the websites of the statistical bureaus of provinces, municipalities and autonomous regions in China can be accessed through the website of the National Bureau of Statistics of China, so the links to the websites of the statistical bureaus of provinces, municipalities and autonomous regions in China are not cited.

**Table 3:** The data sources of input-output indicators.

	Input-Output Indicators	China	Japan	South Korea
**Input indicator 1**	Labor	The Bureau of Statistics of provinces, municipalities and autonomous regions [56]	Portal Site of Official Statistics of Japan [57]	Korean Statistical Information Service [58]
**Input indicator 2**	Capital Stock	National Bureau of Statistics of China [56]	Cabinet Office, Economic and Social Research Institute [59]	Korean Statistical Information Service [58]
**Input indicator 3**	Total Energy Consumption	The Bureau of Statistics of provinces, municipalities and autonomous regions [56]	Agency for Natural Resources and Energy [60]	Korea Energy Statistical Information System [61]
**Desirable output indicator**	GDP	National Bureau of Statistics of China [56]	Cabinet Office, Economic and Social Research Institute [59]	Korean Statistical Information Service [58]
**Undesirable output indicator**	Carbon Dioxide Emission	China Emission Accounts and Datasets (CEADs) [62]	Agency for Natural Resources and Energy [60]	Korea Energy Statistical Information System [61]

Table 4 provides descriptive statistics on the input-output indicators. The statistical items include the mean, standard deviation, minimum value, maximum value, and coefficient of variation. The statistical samples are divided into PLADs of China, PLADs in Japan, and PLADs in South Korea, and a full sample of China, Japan and South Korea. This paper believes that the following three aspects are worth paying attention to:

1. The average value of labor, capital stock, and total energy consumption in Chinese PLADs is 2.87, 2.75, and 2.69 times the average of the full sample. The average GDP as an indicator of desirable output is 1.99 times the average of the full sample, and the average carbon dioxide emission as an indicator of undesirable output is 2.86 times the average of the full sample. The average value of labor, capital stock, and total energy consumption in the PLADs of Japan are 0.11, 0.17 and 0.16 times the average of the full sample, respectively. The average GDP of the output indicator is 0.56 times that of the full sample, and the average value of carbon dioxide emissions is 0.10 times that of the full sample. The average value of labor, capital stock, and total energy consumption in the PLADs of South Korea is 0.16, 0.19 and 0.33 times the average of the full sample. The average GDP is 0.47 times the average of the full sample, and the average of carbon dioxide emissions is 0.21 times the average of the full sample. The multiple of the mean of labor force in Chinese PLADs/the mean of full sample is 26.38 and 17.70 times that of Japan and South Korea, respectively. The multiple of the mean of capital stock in Chinese PLADs/the mean of full sample is 15.91 and 14.56 times that of Japan and South Korea, respectively. The multiple of the mean of total energy consumption in Chinese PLADs/the mean of full sample mean is 16.33 and 8.16 times that of Japan and South Korea. The multiple of the average of GDP in Chinese PLADs/the mean of full sample is 3.56 and 4.23 times that of Japan and South Korea. In addition, the multiple of the mean of carbon dioxide emissions in Chinese PLADs/the mean of full sample is 28.18 times that of Japan and 13.93 times that of South Korea. It can be seen that the multiple of the overall mean of PLADs in China/the mean of full sample is much higher than that of Japan and South Korea in terms of input and undesirable output indicators, while the GDP as an indicator of desirable output is only 3.56 and 4.23 times higher than that of Japan and South Korea. Higher input costs and undesirable outputs, as well as lower desirable outputs, will lead to inefficiencies in a low-carbon economy.
2. The standard deviation, minimum value and maximum value show the data distribution of the PLADs of China, Japan, and South Korea, also the full sample.
3. The coefficient of variation is used as a relative index to measure the degree of dispersion of data, which can compare the degree of dispersion among several samples under variables with the same dimension but greatly different mean values. Statistics show that among the five input-output indicators, the PLADs of China is smaller than Japan and South Korea, indicating that the distribution difference of various input-output indicators among China is smaller than that of Japan and South Korea. The coefficient of variation of Japanese PLADs is larger than that of South Korea in terms of labor force, capital stock, total energy consumption, and GDP. As for carbon dioxide emissions, the coefficient of variation of Japanese PLADs is smaller than that of South Korea, but the difference between the two is small, only 0.04.

### 4.4. Data Pre-Processing

#### 4.4.1. China

The units of input-output indicators of all samples in China, Japan and South Korea need to be consistent. This paper takes Chinese statistical data units as the benchmark, so only the units of samples from Japan and South Korea need to be adjusted. The data pre-processing of the Chinese part mainly goes through the following two steps:

1. The capital stock and GDP collected in this paper are nominal values, which have not been adjusted for inflation and cannot accurately measure the actual level of the indicators. Therefore, this paper uses 2015 as the base period for constant prices and calculates the actual value in other years. The reason for choosing 2015 is that the data of Japan and South Korea on these two indicators are the actual values obtained by deflating 2015 as the base period. To facilitate the calculation, those two indicators in China are also deflated with 2015 as the base period. The specific calculation method is that the data value in 2015 × the GDP deflator in 2016 compared with 2015, and the actual value in 2016 can be obtained. The calculation method is also used for 2017–2019. For the real values in 2014, derived from 2015 data values/the GDP deflator in 2015 compared with 2014, and the same for 2013. Among them, the GDP deflator measures the current price level relative to the price in base year, and the data comes from the website of the National Bureau of Statistics of China [56].
2. China no longer publishes capital stock data after 2018, so the average growth rate of the previous three years is used, that is, the growth rate in 2015 compared with 2014, the growth rate in 2016 compared with 2015, and the growth rate in 2017 compared with 2016. The average growth rate is used as the growth rate of 2018 compared with 2017, to supply data for this indicator in 2018. Take the growth rate of 2016 compared with 2015, the growth rate of 2017 compared with 2016, and the growth rate of 2018 compared with 2017 to calculate the average value. The average growth rate is used as the growth rate of 2019 compared with 2018, and the data requirement for this indicator in 2019 is fulfilled.

#### 4.4.2. Japan

The data pre-processing of the Japanese part mainly goes through the following 6 steps:

1. Convert the unit of labor force from thousand to ten thousand.
2. Complete the missing data of capital stock. In terms of capital stock indicators, Aomori, Fukui, Nara, Okinawa and Tokushima lack data in 2019, so the average growth rate of the previous three years is used, that is, the growth rate in 2016 compared with 2015, the growth rate in 2017 compared with 2016, and the average growth rate in 2018 compared with 2017. The average growth rate is used as the growth rate in 2019 compared with 2018, so as to supply data for this indicator in 2019.
3. Complete the missing data of GDP. In terms of GDP indicators, Aomori, Fukui, Nara, Okinawa, and Tokushima still lack data for 2019.The filling method of the indicator data in 2019 is consistent with the method of the capital stock indicator.
4. Convert the unit of capital stock and GDP. The unit of capital stock and regional gross domestic product, one million yen is converted into one hundred million yuan, and is converted by the standard price in the foreign exchange rate announced by the Bank of China on December 31 of that year. The data comes from the Bank of China [63].
5. Convert the unit of total energy consumption. The unit of total energy consumption, converting trillion Joules to ten thousand tons of coal equivalent. According to the International Energy Agency conversion standard [64], 1 trillion joules = 34.12 tonne(s) of coal equivalent (tce); after conversion to tce, divide by 10,000 to convert to ten thousand tons of coal equivalent.
6. Convert the unit of carbon dioxide emissions. The molecular weight of carbon is 12, and the molecular weight of carbon dioxide is 44. 44/12 = 3.67, which means that 1 ton of carbon can produce about 3.67 tons of carbon dioxide after burning in oxygen. The carbon emissions in Japan (unit: 10^3^ t) is converted into carbon dioxide emissions (unit: ton t), the indicator needs to be multiplied by 3.67 and then divided by 10^3^ to complete the conversion.

#### 4.4.3. South Korea

The data pre-processing of the South Korean part mainly goes through the following five steps:

1. Convert the unit of labor force from thousand to ten thousand.
2. Convert the unit of capital stock and regional GDP indicators. One million won is converted into one hundred million yuan. The standard price in the foreign exchange rate announced by the Bank of China on December 31 of that year is used for conversion. The data comes from the Bank of China [63].
3. Complete the data of Sejong in 2013. Among the indicators of total energy consumption and carbon dioxide emissions, Sejong lacks data in 2013. In 2013, the two indicators of Sejong were collected by Chungcheongnam-do. This paper calculates the growth rate of 2015 compared with 2014, the growth rate of 2016 compared with 2015, and the growth rate of 2017 compared with 2016. The three-year average growth rate is calculated as the growth rate of 2014 compared with 2013, so as to calculate the data of Sejong in 2013, and deduct the corresponding data from Chungcheongnam-do to correct the 2013 data of this indicator for Chungcheongnam-do.
4. Convert the unit of total energy consumption. According to the conversion standard of the International Energy Agency [64], 1 toe= 1.429 tce, and the unit of total energy consumption is 10^3^ tonne(s) of oil equivalent (toe), which is 1429 tce, and divided by 10,000 after conversion to tce. The result obtained is ten thousand tons of coal equivalent.
5. Convert the unit of carbon dioxide emissions. Since there is no published data on carbon dioxide emissions, this paper calculates CO_2_ emissions based on CO_2_ emission factors from the combustion of different energy sources published by the Intergovernmental Panel on Climate Change [65]. First, convert the energy unit, and 1000 toe is converted into 41.87 TJ [64], then multiplied by the CO_2_ emission factor (unit: Kg/TJ) to obtain the kilogram carbon dioxide emission value. According to the standard 1 ton = 1000 Kg, it is converted into ten thousand tons of carbon dioxide emissions.

## 5. Empirical Research

### 5.1. Analysis of Low-Carbon Economic Efficiency Evaluation Results

This paper uses Matlab to construct an SBM model with undesirable output based on global covariance. Firstly, this paper measures the low-carbon economic efficiency values of 94 PLADs of China, Japan, and South Korea from 2013 to 2019. The measurement result of 1 indicates that the low-carbon economic efficiency of that division is effective relative to others, and the lower the result, the lower the low-carbon economic efficiency of that division. Further, the PLADs of China, Japan, and South Korea whose average low-carbon economic efficiency does not reach 1 are selected for redundancy analysis of input indicators and undesirable output indicators, and for deficiency analysis of desirable output indicators.

As can be seen from Table 5, the average low-carbon economic efficiency in China from 2013 to 2019 was 0.094, which was far lower than the effective value of 1, but the change trend showed a steady rise. Guangdong and Jiangsu both achieved efficiency values of 1.000 in 2019, and the eastern PLADs such as Beijing, Shanghai and Fujian ranked in the forefront of the carbon emission efficiency value, while the average efficiency of Heilongjiang, Jilin and Liaoning in the northeast region ranked at the lower end for China, even not reaching 0.1. Overall, Chinese low-carbon economy efficiency is higher in the eastern region, followed by the western region, lower in the central region, and the lowest in the northeastern region. The overall low-carbon economic efficiency is lower, and the efficiency values of most PLADs are even less than 0.1. There is much room for improvement.

From the input indicators in Table 5, non-DEA effective PLADs of China have a large degree of input redundancy, indicating that the input indicators have low utilization efficiency. Among them, the input redundancy rate of total energy consumption is the largest. Except for Guangdong and Jiangsu which reached the efficiency value of 1 in 2019, the input redundancy of total energy consumption in other divisions is close to 90%. These divisions need to significantly reduce energy consumption to improve their low-carbon economic efficiency. There is also redundancy in the labor and capital stock, which greatly affects the efficiency. From the perspective of output indicators, the deficiency rate of the desirable output GDP is 0, that is, there is no need to further pursue high-speed growth of GDP to achieve high low-carbon economic efficiency. There is a problem of excessive CO_2_ emissions. Except for Guangdong and Jiangsu, the inefficiency of CO_2_ emissions in other divisions is at a high redundancy level of more than 90%, that is, excessive CO_2_ emissions have a great effect on urban low-carbon economic efficiency.

As shown in Table 6, the average low-carbon economic efficiency in Japan from 2013 to 2019 reaches 0.533, and the change trend shows the characteristics of rising fluctuations. Among them, Tokushima, Tokyo, and Nara achieved an efficiency value of 1.000 in 2019. From the 7-year data, their average low-carbon economic efficiency is also in the top 5. Another two divisions in the top five are Tottori and Yamanashi, with efficiency values of 0.932 and 0.837, while Ehime, Yamaguchi, Hiroshima, Oita and Okayama are the last 5 divisions in the 7-year average ranking of low-carbon economic efficiency. Compared with China, the low-carbon economic efficiency value of Japan has been greatly improved, but there are still large differences between divisions, and there is room for further improvement.

Judging from the redundancy and deficiency of Japanese input-output indicators, the redundancy rate of total energy consumption is the largest, followed by the redundancy rate of labor, and the redundancy rate of capital stock is the lowest. Several divisions with a 7-year average of low-carbon economic efficiency rank among the top 9, including Tokushima, Tottori, Nara, Saga, Shimane and Yamagata, and the capital stock redundancy rate of these 6 divisions is 0. In addition, the capital stock redundancy rate of Kagoshima is also 0, and this indicator does not need to be further optimized. The remaining input indicators for the above divisions and three input indicators for the remaining divisions still exist, so there is room for further improvement in the resource allocation efficiency. The desirable output GDP of the 47 PLADs is not insufficient, but the CO_2_ emissions are a significant factor affecting the low-carbon economic efficiency of most divisions. The redundancy rate of CO_2_ emissions in Chiba, Yamaguchi, Oita and Okayama has reached more than 90%.

As shown in Table 7, the average low-carbon economic efficiency of South Korea from 2013 to 2019 is 0.337, and the overall change showed a trend of slight recovery after decline. The average low-carbon economic efficiency of Sejong in the seven-year period ranks first within the country, reaching 0.796, which is close to the efficiency value of 1. In contrast, in other PLADs, the average low-carbon economic efficiency is lower than 0.5, showing great differences between regions. The overall low-carbon economic efficiency value in South Korea is at a low level, and the efficiency values in most divisions have not reached the effective value.

The redundancy of labor and total energy consumption has a great impact on the low-carbon economic efficiency. The redundancy rate of capital stock is relatively small, and the redundancy rate of capital stock in Busan is 0. Ulsan, Chungcheongnam-do, and Jeollanam-do have a redundancy rate of over 90% in total energy consumption, and Daegu, Busan, Jeollabuk-do, Gangwon-do, and Incheon have over 70% redundancy in labor. There is still much room for improvement in terms of input factors. The deficiency rate of the desirable output GDP is 0, and the undesirable output CO_2_ emissions have a great impact on the low-carbon economic efficiency of South Korea. Among them, the CO_2_ emissions of Ulsan, Gyeongsangbuk-do, Incheon, Chungcheongnam-do and Jeollanam-do have an impact of more than 90% on the low-carbon economy efficiency.

### 5.2. Analysis of Low-Carbon Economic Efficiency Prediction Results

In this paper, the efficiency value sequences of 94 PLADs in the two groups of China, Japan and South Korea from 2013 to 2017 and 2014 to 2018 are used as the input of the training samples, and the efficiency values of 2018 and 2019 are used as the output of the training samples, forming a total of 188 training samples. Based on the training samples, this paper uses 10 mainstream machine learning models combined with the GridSearchCV method to find the model with the best prediction effect; the coefficient of determination, R-squared, is used as the evaluation index to measure the prediction effect. According to Table 8, the model with the best prediction effect is the XGBoost model, and the parameter configuration of all models is shown in Table A1 of Appendix A. Then, we use the XGBoost model to predict the low-carbon economic efficiency value of the PLADs of China, Japan and South Korea from 2020 to 2024. The codes of all models are developed in Python language and run using PyCharm 11 software.

The prediction results for China are shown in Table 9. The low-carbon economic efficiency value of most divisions will decline slightly from 2020 to 2021, but the decline is limited, and it will rise steadily after 2022. The PLADs where the low-carbon economic efficiency will improve from 2020 to 2024 covers most of the eastern coastal central provinces, as well as a small number of western provinces, including Guangdong, Jiangsu, Shanghai, Fujian, Hubei, Shaanxi, Jiangxi, Hunan, Anhui, Yunnan, Sichuan, and Shandong. Among them, the increase in Guangdong, Jiangsu and Fujian is larger than that of other provinces, and for Jiangsu the increase is from 0.901 to 0.978, which is close to the effective value of 1. As the developed provinces in the eastern coastal area, they are in the forefront of the country in terms of technology and market for low-carbon economic development. Correspondingly, the efficiency has also achieved steady growth. It is worth noting that the western region, as the industrial area of “high-carbon economy” in the past, has benefited from the vigorous development of new energy in Yunnan and Sichuan. The divisions where the low-carbon economic efficiency drops significantly cover most of the western provinces, such as Gansu, Guizhou, Qinghai, Guangxi and other provinces. Most of the provinces in the western region of China adopt an extensive development model. Especially after the industrial transfer from the eastern provinces to the western provinces, a large number of industrial businesses have settled in the western region. At the same time, they also produced a large amount of CO_2_ emissions. Excessive undesirable output has become one of the bottlenecks restricting the sustainable development of the western provinces. The low-carbon economic efficiency in Northeast China and a few eastern provinces do not change significantly. Among them, the three provinces in Northeast China are limited by geographical location and resource conditions, and the slow transition process results in unsmooth development of a low-carbon economy. As the leading provinces of economic development, Beijing, Tianjin and Hebei have stagnated in the development of a low-carbon economy. The reason is that the products of these provinces are mainly steel, chemicals, building materials, automobiles, etc., which are the main heavy industries, and the corresponding energy consumption intensity is relatively large.

**Figure 1:**
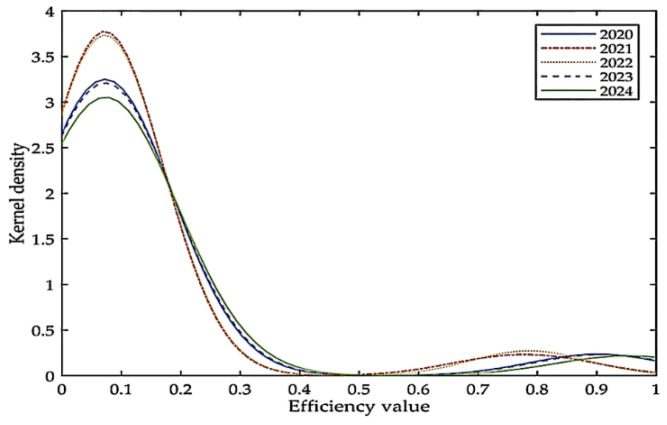
Kernel density estimates of low-carbon economic efficiency values in China.

In order to further examine the dynamic evolution and regional differences of low-carbon economic efficiency in each PLAD from 2020 to 2024, this paper analyzes it through kernel density estimation. Figure 1 shows the kernel density estimation of low-carbon economic efficiency values in 30 Chinese PLADs. From 2020 to 2024, the peak height of efficiency values drops significantly and the range of change shifts to the right, indicating that the kernel density corresponding to the peak decreases and the efficiency value increases. In other words, the divisions whose low-carbon economic efficiency value is less than 0.1 will decrease, and the efficiency values of most divisions will show an upward trend. The second wave peak shows a trend of moving to the right as a whole, indicating that the efficiency values of the divisions that were previously at a high level will be further improved. In general, the low-carbon economic efficiency value of China will increase, and the number of PLADs that achieve effective condition will also increase.

Table 10 shows the prediction results of low-carbon economic efficiency values for 47 PLADs in Japan from 2020 to 2024. The overall efficiency in Japan shows a steady upward trend, with the national average efficiency rising from 0.558 to 0.576. More than half of the divisions will achieve the improvement of efficiency. Among them, the eight divisions that will reach the efficiency value of 0.900 in 2024 include Tokushima, Tottori, Tokyo, Yamanashi, Nara, Saga, Kochi and Shimane. Among the above divisions, except Tokyo, which is the leading city in domestic economic development, the economic development of the other divisions is at a relatively low level. Most of them are dominated by agricultural development, so CO_2_ emissions are relatively low, and the low-carbon economic efficiency values are high. Divisions that do not achieve efficiency growth include Daban, Kyoto, Gunma, Nagano and other cities in the forefront of economic development, as well as low-income areas represented by Akita, Wakayama, and Tottori. From the perspective of energy consumption in the divisions, the coal and crude oil used in most low-efficiency divisions are significantly higher than the national average, while most of the high-efficiency divisions use clean energy represented by nuclear energy, so there is a significant imbalance. For the overall efficiency value distribution, the low-carbon economic efficiency values of PLADs of Japan are relatively high, which also shows that clean energy is now the main feature of the Japanese energy system.

**Figure 2:**
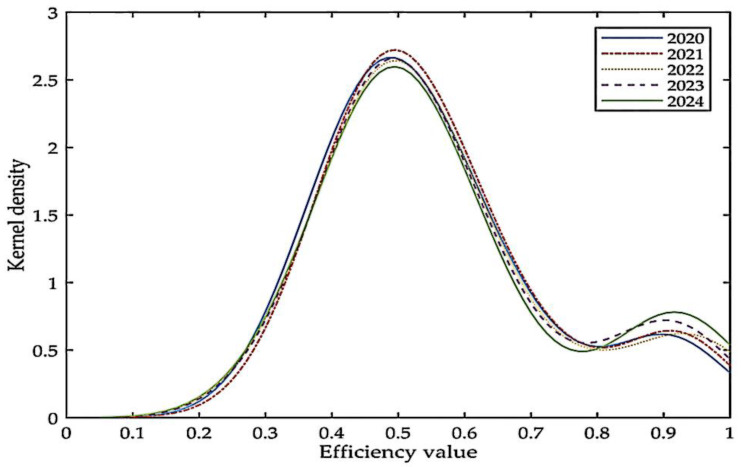
Kernel density estimates of low-carbon economic efficiency values in Japan.

Further analysis of the kernel density estimation of low-carbon economic efficiency prediction in Japan is shown in Figure 2. The peak height of the density curve in 2020–2024 has a slight decrease, that is, the PLAD near the peak of 0.5 has a small decrease. The second peak increases significantly in 2024, that is, the number of divisions near the peak value of 0.9 increases. Overall, low-carbon economic efficiency in Japan shows a steady upward trend; in particular, the PLADs with high efficiency values increase significantly compared to 2020.

The prediction results of the low-carbon economic efficiency values in South Korea from 2020 to 2024 are shown in Table 11. The average low-carbon economic efficiency of South Korea rises steadily, from 0.340 in 2020 to 0.353, and more than half of the PLADs achieve an increase in the efficiency value. Among them, the efficiency value of Sejong increases from 0.782 to 0.963, which is close to the effective value of 1. As the administrative capital of South Korea, Sejong always pays attention to green development. It is committed to the development of green agriculture, clean energy, IT, biotechnology and other cutting-edge urban industries. It is a model city for carbon reduction in South Korea. Jeju, Daejeon, Chungcheongbuk-do and other divisions also achieve low-carbon economic efficiency improvements. Jeju, as a test area for electric vehicles in South Korea, is supported by various government policies and hopes to make it a net zero carbon island. Daejeon is the science and technology center of South Korea, and it mainly develops the tertiary industry, while Chungcheongbuk-do is dominated by agriculture. The CO_2_ emissions of these industries are relatively low, which is conducive to the low-carbon economic development in these divisions. Divisions with reduced low-carbon economic efficiency, represented by Incheon and Gyeonggi-do, all face the same problem. The expansion of urbanized coastlines leads to an increase in commuting distances and other changes, which further increase energy consumption and CO_2_ emissions. Industrial cities such as Daegu and Ulsan will reduce their low-carbon economic efficiency values due to a large amount of petrochemical energy consumption.

**Figure 3:**
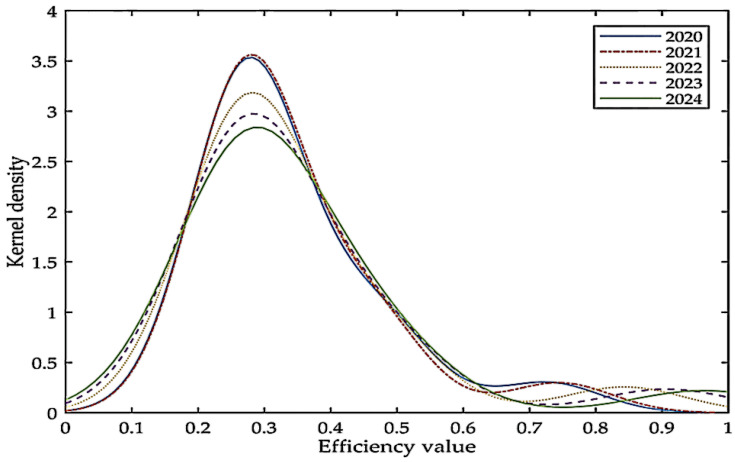
Kernel density estimates of low-carbon economic efficiency values in South Korea.

Figure 3 shows the estimated kernel density of the low-carbon economy efficiency prediction in South Korea. The peak in 2024 drops significantly and the change interval becomes larger, indicating that the number of provincial-level administrative divisions with an efficiency value of 0.3 will decrease. The efficiency of some divisions will increase, but at the same time the divisions with efficiency values less than 0.3 also increase. The second peak obviously shows a right-shift trend, that is, the efficiency value of the divisions with higher efficiency value will further increase. The overall low-carbon economic efficiency value in South Korea is on the rise, but it is worth noting that the number of divisions with lower efficiency values will also increase.

### 5.3. Discussion

This paper measures the low-carbon economic efficiency values of 94 PLADs in China, Japan, and South Korea, and we find that the 7-year average values from 2013 to 2019 show Japan > South Korea > China, which is consistent with the results presented in the related research literature [24,66]. This paper further investigates the low-carbon economic efficiency values of 94 PLADs in China, Japan, and South Korea, which is different from previous studies that mainly used national-level data to measure the low-carbon economic efficiency. This paper uses labor force, capital stock, total energy consumption, regional GDP, and carbon dioxide emissions data of 94 PLADs to measure their low-carbon economic efficiency, and it enriches the study of low-carbon economic efficiency of PLADs in different countries.

Based on the measurement of historical low-carbon economic efficiency values, this paper uses 10 machine learning models combined with the GridSearchCV method to predict the low-carbon economic efficiency of PLADs in China, Japan and South Korea. According to the coefficient of determination R2 of the model prediction effect, all the 10 machine learning models have good prediction effect. In this paper, the XGBoost model with the best prediction effect is selected to predict the low-carbon economic efficiency values from 2020 to 2024, which makes up for the shortcoming that DEA can only evaluate the historical efficiency values of DMUs. The efficiency prediction model formed by combining the DEA method and machine learning method provides a new method for efficiency evaluation and prediction, and the prediction results will provide a reference and basis for scientific decision making in relevant countries.

## 6. Conclusions and Recommendations

The main conclusions of this paper are as follows:

1. From the evaluation results, Japan had the highest average national low-carbon economic efficiency from 2013 to 2019, followed by South Korea and China. The low-carbon economic efficiency of Japan from 2013 to 2019 has steadily improved, with an average value of 0.533. The low-carbon economic efficiency value of Tokyo, Tokushima and Nara reached an effective value of 1.000 in 2019, and 20 of 47 PLADs reached the national average efficiency value and above. From 2013 to 2019, the low-carbon economy efficiency value of South Korea decreased, with an average value of 0.337. From the perspective of the PLADs, none reached the effective value of 1.000, and only 5 of 17 PLADs in the country reached the national average. The low-carbon economic efficiency of China from 2013 to 2019 shows a clear upward trend, with an average value of 0.094. Guangdong and Jiangsu achieved an effective value of 1 in 2019. Among the 30 PLADs in the country, only 4 divisions reached the national average efficiency value. From a country perspective, the low-carbon economic efficiency of China, Japan and South Korea show obvious regional differences. From a domestic perspective, only a small number of PLADs in China have reached the national average efficiency.
2. From the perspective of input-output indicators, the input indicators of China have serious problems of excessive input and low utilization efficiency. In China, the redundancy rate of total energy consumption is the largest, and most of the PLADs have reached 90%. There are also problems of excessive investment in labor and capital stock. Most divisions in China have a high redundancy rate of CO_2_ emission, which may reach more than 90%, and there is much room for improvement. Among the input indicators in Japan, the redundancy rate of total energy consumption is the largest, followed by redundancy rate of labor input, and redundancy rate of capital stock is the lowest. Undesirable output, CO_2_ emission, is a significant factor affecting the low-carbon economic efficiency of most Japanese PLADs. The labor, total energy consumption, and carbon dioxide emissions of Korea have a great impact on the low-carbon economic efficiency.
3. From the prediction results, the overall low-carbon economic efficiency value of China will drop slightly from 2020 to 2021, and will rise steadily after 2022. The number of divisions that achieve low-carbon economic efficiency improvement is increasing year by year. Among them, the efficiency value of the divisions in the coastal areas where low-carbon technologies and markets are more developed will increase greatly, while the low-carbon economic efficiency values of the divisions in the western and northeastern regions, which are subject to regional resources and industrial structure, will decrease slightly. The national low-carbon economic efficiency value of Japan will show a steady upward trend from 2020 to 2024, and the average value will rise to 0.576 in 2024. The number of high-efficiency PLADs will increase significantly. The low-carbon efficiency of the divisions in Japan shows non-equilibrium characteristics, and divisions that use clean energy represented by nuclear energy have significantly higher efficiency values than the divisions that use coal and crude oil. The low-carbon economic efficiency value of South Korea from 2020 to 2024 will increase year by year, and the overall average value will reach 0.353 in 2024. Both the number of high-efficiency and low-efficiency PLADs will increase, and the GDP will continue to expand. In South Korea, the efficiency values of divisions that are dominated by the primary and tertiary industries are generally high, while the efficiency values of some industrial cities show a clear downward trend.

According to the above research findings, the low-carbon economic efficiency of China, Japan, and South Korea show a steady upward trend from 2020 to 2024, but there are still problems such as large regional differences and low resource allocation efficiency. The three countries share a common low-carbon economy goal, have many common interests, and have a wide range of cooperation prospects, and this paper proposes the following recommendations in light of the current situation of low-carbon economic development in each country.

1. Focus on inter-regional differences and make full use of the endowment advantages of each country. From the evaluation results, it can be seen that there are large differences in the low-carbon economic efficiency values between China and Japan and South Korea, and there is still a significant gap in the predicted values after five years. In response to the different development situations of the three countries, it is necessary to make full use of their own advantages to develop differentiated low-carbon economic development strategies. China is a major energy producer and consumer, especially in the development of natural gas, solar energy, wind energy and other clean and new energy sources, and has a unique advantage. China should seize the opportunity to take advantage of its vast territory and abundant energy resources to increase the research, development and promotion of new energy technologies. Japan and South Korea are small energy producing and consuming countries, with high external energy dependence. As developed countries, both these two countries have high domestic carbon reduction costs. Japan and South Korea have the advantage of leading international energy-saving and emission reduction technologies, have mastered key technologies in waste treatment, soot desulfurization and other fields, and have accumulated rich experience in the field of energy utilization. China, Japan and South Korea have strong complementarities in energy-saving technologies and energy resources, and it is necessary for the three countries to form a collaboration on emission reduction to achieve emission reduction outside at a lower cost.
2. Optimize the energy structure and drive industrial development with new energy sources. Based on the empirical results of this paper, a high redundancy rate of CO_2_ emissions is common in China, Japan and South Korea, which significantly affects the improvement of low-carbon economic efficiency in each country, and they need to further adjust the energy structure and reduce the proportion of fossil energy consumption. The energy consumption of China is dominated by coal, and its energy structure, which is determined by resource endowment, is the root cause of the high energy carbon intensity in China. In the energy structure of Japan, the reliance on oil is decreasing, and the use of natural gas and nuclear energy is increasing. Japan is also focusing on clean technologies for fossil energy in order to reduce the environmental problems caused by fossil energy. Similar to Japan, South Korea is focusing on diversification of energy outcomes by developing nuclear energy and importing liquefied natural gas to meet energy demand. Although China, Japan and South Korea are in different processes of restructuring their energy institutions, renewable energy is the focus of attention in all three countries. China is currently a global leader in the field of renewable energy, especially in the solar photovoltaic, wind power, hydroelectric power and other industries. While Japan and South Korea have world-class R&D and technological advantages, their domestic resources and environment place a non-negligible constraint on the development of the low-carbon economy. China, Japan and South Korea should effectively integrate technical and new energy resources and cooperate with each other, so they can achieve a diverse low-carbon energy portfolio and obtain multi-win results.
3. Focus on international cooperation and drive regional cooperation with business cooperation. From the prediction results, the low-carbon economic efficiency in the eastern coastal regions of China, in Japan and South Korea, with developed science and technology industries is generally higher; this is inseparable from international cooperation. Especially in the context of addressing climate change, whether from the perspective of national interests or global environmental interests, regional environmental cooperation among China, Japan, and South Korea will play a positive role in promoting national economic development and achieving global common interests. However, influenced by regional politics, rights, and perceptions, there are also difficulties in regional environmental cooperation between the governments of China, Japan, and South Korea. Thus, business-to-business cooperation is expected to be the main vehicle for regional environmental cooperation. At present, Japan has developed corresponding projects in China through the Clean Development Mechanism (CDM). Enterprise cooperation has become a major component of China-Japan low-carbon economic cooperation, and is expected to become the main body of regional low-carbon economic cooperation among China, Japan and South Korea. In the future, enterprises in these three countries should continue to deepen their cooperation in the fields of low-carbon technology transformation, green direct investment and low-carbon technology trading, and then promote the low-carbon economic development worldwide and build a community of green destiny for mankind.

Finally, we may note the limitations of this study and also the direction of our further research in the future. (1) This paper has studied the low-carbon economic efficiency of the PLADs of China, Japan and South Korea using macro data, and the suggestions made are mostly at the macro level. In fact, applying evaluation and prediction models to micro subjects such as industries and enterprises is a future research direction. (2) The construction of the evaluation index system uses structured data and lacks research on unstructured data. For example, it could utilize text data from mainstream social networking platforms. By using natural language techniques, researchers could analyze people’s emotional perceptions of the regional ecological environment and construct a low-carbon economic efficiency evaluation index system. (3) Although this paper achieved good model prediction results in the empirical study, the prediction accuracy of the machine learning model can still be further improved by applying feature engineering.

## Figures and Tables

**Table 2:** Low-carbon economic efficiency evaluation index system.

	Input-Output Indicator	Measurement	Unit
**Input indicator 1**	Labor	Number of employed population at the end of the year in PLADs	ten thousand people
**Input indicator 2**	Capital Stock	Real Gross Fixed Capital in PLADs (deflated with 2015 as the base period)	RMB 100 Million Yuan
**Input indicator 3**	Total Energy Consumption	Total energy consumption in PLADs	Ten thousand ton of Standard Coal Equivalent
**Desirable output indicator**	GDP	Real GDP in PLADs (deflated with 2015 as the base period)	RMB 100 Million Yuan
**Undesirable output indicator**	Carbon Dioxide Emission	Carbon dioxide emissions in PLADs	Million Tons

**Table 4:** Descriptive statistics of input-output indicators.

Input-Output Indicators	Sample	Mean	Standard Deviation	Minimum	Maximum	Coefficient of Variation
Labor (ten thousand person)	PLADs of China	2755.57	1772.51	314.20	6995.00	0.64
	PLADs of Japan	104.45	149.05	9.91	1071.46	1.43
	PLADs of South Korea	155.69	165.87	6.01	704.30	1.07
	Full Sample	959.82	1590.51	6.01	6995.00	——
Capital Stock (RMB 100 Million Yuan)	PLADs of China	26,144.13	19,876.77	2045.71	97,440.07	0.76
	PLADs of Japan	1643.43	1981.59	265.66	13,826.07	1.21
	PLADs of South Korea	1796.19	1850.10	291.74	9813.82	1.03
	Full Sample	9490.43	16,076.81	265.66	97,440.07	——
Total Energy Consumption (Ten thousand ton of Standard Coal Equivalent)	PLADs of China	15,205.01	8863.27	1720.33	41,390.00	0.58
	PLADs of Japan	931.17	945.00	143.38	4810.90	1.01
	PLADs of South Korea	1862.99	1722.99	61.94	5995.70	0.92
	Full Sample	5655.17	8300.57	61.94	41,390.00	——
GDP (RMB 100 Million Yuan)	PLADs of China	24,927.67	19,916.15	1702.01	97,953.88	0.80
	PLADs of Japan	7001.23	10,155.97	909.19	72,968.51	1.45
	PLADs of South Korea	5886.29	6578.05	395.10	27,885.23	1.12
	Full Sample	12,520.79	16,058.50	395.10	97,953.88	——
Carbon Dioxide Emission (Million tons)	PLADs of China	384.67	320.51	44.05	1700.04	0.83
	PLADs of Japan	13.65	15.83	1.19	83.67	1.16
	PLADs of South Korea	27.62	33.04	0.41	120.45	1.20
	Full Sample	134.59	249.78	0.41	1700.04	——

**Table 5:** Evaluation results of low carbon economic efficiency value in China from 2013 to 2019.

	2013	2015	2017	2019	7-Year Average	Redundancy Rate of Labor	Redundancy Rate of Capital Stock	Redundancy Rate of Total Energy Consumption	Deficiency Rate of Regional GDP	Redundancy Rate of CO_2_ Emissions
Guangdong	0.082	0.148	0.527	1.000	0.417	52.63%	47.04%	56.51%	0.00%	58.42%
Jiangsu	0.086	0.095	0.424	1.000	0.366	50.17%	54.16%	63.81%	0.00%	69.80%
Beijing	0.115	0.122	0.130	0.138	0.126	77.11%	79.38%	88.42%	0.00%	91.34%
Shanghai	0.100	0.108	0.117	0.125	0.113	78.31%	79.91%	91.91%	0.00%	95.11%
Fujian	0.079	0.082	0.094	0.100	0.088	87.60%	80.65%	92.57%	0.00%	96.64%
Zhejiang	0.080	0.085	0.090	0.094	0.087	87.10%	81.08%	93.02%	0.00%	96.99%
Hainan	0.087	0.086	0.087	0.087	0.087	92.75%	80.09%	88.75%	0.00%	96.05%
Chongqing	0.075	0.079	0.089	0.089	0.083	89.27%	81.00%	92.99%	0.00%	95.93%
Hubei	0.069	0.073	0.079	0.084	0.076	90.56%	81.82%	93.77%	0.00%	96.63%
Shaanxi	0.070	0.073	0.077	0.080	0.075	90.38%	81.26%	94.58%	0.00%	98.97%
Jiangxi	0.070	0.073	0.077	0.080	0.075	92.71%	81.11%	92.81%	0.00%	96.82%
Hunan	0.067	0.071	0.077	0.083	0.074	91.66%	81.51%	93.70%	0.00%	96.74%
Anhui	0.069	0.072	0.076	0.078	0.074	93.78%	79.74%	93.42%	0.00%	98.15%
Yunnan	0.067	0.070	0.073	0.075	0.071	94.24%	79.27%	94.78%	0.00%	97.26%
Sichuan	0.065	0.068	0.072	0.076	0.070	92.94%	81.09%	94.82%	0.00%	96.63%
Henan	0.063	0.066	0.070	0.074	0.068	93.68%	81.30%	94.54%	0.00%	98.03%
Shandong	0.062	0.065	0.070	0.076	0.068	90.34%	83.70%	95.46%	0.00%	98.68%
Guizhou	0.062	0.064	0.068	0.070	0.066	93.95%	81.12%	95.44%	0.00%	98.64%
Xinjiang	0.064	0.065	0.066	0.067	0.066	91.84%	81.25%	97.46%	0.00%	99.02%
Tianjin	0.064	0.067	0.062	0.063	0.065	89.23%	87.46%	94.54%	0.00%	97.15%
Guangxi	0.059	0.062	0.064	0.065	0.063	94.19%	83.40%	94.55%	0.00%	97.41%
Qinghai	0.060	0.061	0.063	0.065	0.062	93.91%	83.41%	95.14%	0.00%	95.33%
Gansu	0.059	0.060	0.062	0.063	0.061	95.51%	81.61%	95.62%	0.00%	98.08%
Shanxi	0.057	0.059	0.060	0.064	0.060	93.17%	82.41%	97.55%	0.00%	99.70%
Hebei	0.056	0.058	0.060	0.063	0.059	93.06%	83.43%	97.02%	0.00%	98.68%
Inner Mongolia	0.054	0.056	0.061	0.064	0.059	89.99%	86.26%	97.49%	0.00%	99.44%
Ningxia	0.058	0.058	0.058	0.059	0.058	94.88%	82.60%	96.31%	0.00%	98.78%
Liaoning	0.053	0.056	0.058	0.063	0.057	90.73%	86.49%	96.76%	0.00%	98.87%
Jilin	0.051	0.054	0.059	0.060	0.056	92.69%	87.72%	94.63%	0.00%	98.18%
Heilongjiang	0.053	0.054	0.057	0.060	0.056	93.65%	85.38%	96.01%	0.00%	98.74%
China	0.069	0.074	0.101	0.139	0.094	88.40%	80.22%	92.15%	0.00%	95.21%

**Table 6:** Evaluation results of low carbon economic efficiency value in Japan from 2013 to 2019.

	2013	2015	2017	2019	7-Year Average	Redundancy Rate of Labor	Redundancy Rate of Capital Stock	Redundancy Rate of Total Energy Consumption	Deficiency Rate of Regional GDP	Redundancy Rate of CO_2_ Emissions
Tokushima	1.000	1.000	0.844	1.000	0.939	6.17%	0.00%	4.04%	0.00%	6.30%
Tottori	0.856	1.000	0.833	0.955	0.932	8.02%	0.00%	9.34%	0.00%	2.49%
Tokyo	1.000	0.758	0.848	1.000	0.875	19.35%	0.08%	7.67%	0.00%	9.59%
Yamanashi	0.700	0.829	0.904	0.907	0.837	10.23%	1.39%	11.52%	0.00%	21.85%
Nara	0.623	0.700	0.697	1.000	0.779	25.39%	0.00%	18.07%	0.00%	21.38%
Saga	0.645	0.740	0.741	0.751	0.715	25.72%	0.00%	23.77%	0.00%	33.73%
Kochi	0.599	0.681	0.679	0.733	0.709	14.46%	0.05%	24.28%	0.00%	48.90%
Shimane	0.601	0.664	0.736	0.647	0.676	34.00%	0.00%	24.22%	0.00%	38.95%
Yamagata	0.570	0.634	0.684	0.675	0.644	38.84%	0.00%	27.97%	0.00%	41.84%
Nagasaki	0.577	0.603	0.634	0.638	0.625	24.51%	0.76%	37.89%	0.00%	52.88%
Fukui	0.533	0.648	0.571	0.600	0.604	25.73%	1.24%	42.17%	0.00%	54.83%
Kyoto	0.534	0.556	0.618	0.622	0.577	23.67%	1.43%	49.73%	0.00%	60.67%
Nagano	0.439	0.552	1.000	0.514	0.571	21.14%	12.05%	49.18%	0.00%	61.88%
Shiga	0.494	0.586	0.603	0.585	0.559	14.54%	9.14%	54.65%	0.00%	64.87%
Ishikawa	0.523	0.579	0.613	0.551	0.556	35.35%	3.20%	45.24%	0.00%	59.65%
Gunma	0.491	0.559	0.590	0.594	0.547	10.71%	14.78%	54.96%	0.00%	68.08%
Tochigi	0.515	0.529	0.622	0.589	0.545	17.08%	6.72%	56.95%	0.00%	68.77%
Kagawa	0.518	0.521	0.551	0.562	0.542	32.03%	7.13%	45.54%	0.00%	64.74%
Kagoshima	0.487	0.498	0.545	0.599	0.541	37.32%	0.00%	49.72%	0.00%	62.58%
Okinawa	0.530	0.523	0.543	0.624	0.537	42.01%	1.84%	44.65%	0.00%	63.21%
Toyama	0.402	0.530	0.504	0.586	0.510	22.49%	7.74%	61.87%	0.00%	72.17%
Akita	0.499	0.532	0.508	0.539	0.509	45.52%	1.69%	51.46%	0.00%	63.56%
Miyagi	1.000	0.396	0.411	0.477	0.507	24.79%	23.30%	56.87%	0.00%	67.54%
Miyazaki	0.499	0.464	0.557	0.508	0.507	35.76%	6.43%	54.14%	0.00%	67.89%
Kumamoto	0.507	0.516	0.488	0.504	0.507	28.09%	8.57%	57.67%	0.00%	70.68%
Hokkaido	0.434	0.402	0.398	0.473	0.506	32.65%	1.79%	66.45%	0.00%	74.55%
Wakayama	0.413	0.424	0.432	0.426	0.498	19.35%	23.00%	62.92%	0.00%	72.73%
Shizuoka	0.467	0.470	0.473	0.499	0.471	33.28%	14.08%	62.40%	0.00%	69.62%
Gifu	0.409	0.466	0.502	0.469	0.463	30.59%	14.42%	64.82%	0.00%	74.26%
Fukushima	0.463	0.437	0.488	0.478	0.461	22.33%	27.47%	60.74%	0.00%	73.86%
Osaka	0.438	0.430	0.484	0.489	0.457	34.33%	10.90%	65.70%	0.00%	76.24%
Saitama	0.425	0.427	0.439	0.482	0.453	40.10%	16.38%	60.78%	0.00%	69.00%
Iwate	0.444	0.436	0.479	0.496	0.452	26.06%	27.11%	59.68%	0.00%	76.45%
Niigata	0.411	0.417	0.438	0.475	0.440	29.18%	18.77%	68.72%	0.00%	77.46%
Aomori	0.423	0.457	0.414	0.419	0.432	55.39%	15.87%	54.40%	0.00%	69.04%
Aichi	0.428	0.382	0.421	0.445	0.418	32.30%	21.14%	69.60%	0.00%	82.23%
Fukuoka	0.432	0.378	0.413	0.426	0.408	39.34%	17.08%	70.46%	0.00%	83.11%
Hyogo	0.403	0.370	0.399	0.438	0.407	30.43%	19.15%	75.20%	0.00%	86.96%
Ibaraki	0.374	0.374	0.441	0.415	0.397	22.89%	22.32%	82.28%	0.00%	89.76%
Mie	0.420	0.413	0.375	0.365	0.387	37.19%	12.32%	82.43%	0.00%	89.16%
Kanagawa	0.378	0.373	0.367	0.406	0.383	40.27%	15.76%	78.68%	0.00%	87.87%
Chiba	0.403	0.379	0.403	0.395	0.379	20.63%	22.26%	89.71%	0.00%	94.68%
Ehime	0.360	0.370	0.403	0.367	0.370	45.00%	21.31%	75.98%	0.00%	84.12%
Yamaguchi	0.372	0.366	0.375	0.372	0.369	30.21%	20.45%	87.28%	0.00%	93.27%
Hiroshima	0.358	0.364	0.383	0.346	0.367	34.26%	27.19%	79.16%	0.00%	89.99%
Oita	0.375	0.357	0.397	0.331	0.358	44.84%	10.41%	87.37%	0.00%	93.27%
Okayama	0.337	0.341	0.342	0.338	0.340	37.38%	23.87%	88.69%	0.00%	94.21%
Japan	0.513	0.520	0.544	0.556	0.533	28.96%	10.86%	54.40%	0.00%	64.91%

**Table 7:** Evaluation results of low carbon economic efficiency value in South Korea from 2013 to 2019.

	2013	2015	2017	2019	7-Year Average	Redundancy Rate of Labor	Redundancy Rate of Capital Stock	Redundancy Rate of Total Energy Consumption	Deficiency Rate of Regional GDP	Redundancy Rate of CO_2_ Emissions
Sejong	1.000	0.772	0.696	0.686	0.796	27.87%	6.39%	15.96%	0.00%	10.35%
Jeju	0.517	0.450	0.381	0.427	0.442	66.41%	4.32%	51.33%	0.00%	69.94%
Seoul	0.439	0.420	0.439	0.468	0.436	54.73%	3.15%	66.64%	0.00%	68.56%
Gwangju	0.412	0.434	0.423	0.438	0.426	69.99%	0.00%	62.25%	0.00%	62.33%
Daejeon	0.377	0.368	0.374	0.377	0.374	67.41%	8.29%	70.29%	0.00%	74.90%
Daegu	0.325	0.309	0.331	0.343	0.327	76.41%	26.70%	67.46%	0.00%	64.14%
Ulsan	0.335	0.327	0.309	0.318	0.324	31.02%	31.40%	93.58%	0.00%	96.29%
Busan	0.299	0.313	0.303	0.305	0.304	70.39%	27.97%	74.70%	0.00%	78.32%
Gyeongsangnam-do	0.295	0.281	0.278	0.302	0.290	64.98%	37.83%	76.61%	0.00%	77.31%
Chungcheongbuk-do	0.272	0.281	0.268	0.281	0.277	64.08%	42.76%	76.47%	0.00%	80.82%
Gyeonggi-do	0.278	0.263	0.262	0.278	0.270	62.77%	41.27%	81.56%	0.00%	82.23%
Jeollabuk-do	0.245	0.245	0.282	0.287	0.263	76.28%	40.96%	75.05%	0.00%	73.02%
Gyeongsangbuk-do	0.245	0.244	0.247	0.259	0.251	59.17%	40.25%	89.85%	0.00%	94.55%
Gangwon-do	0.246	0.237	0.251	0.260	0.249	72.66%	44.87%	76.71%	0.00%	83.71%
Incheon	0.250	0.245	0.244	0.248	0.245	70.37%	37.55%	85.23%	0.00%	90.19%
Chungcheongnam-do	0.241	0.226	0.228	0.255	0.237	50.21%	50.57%	93.84%	0.00%	96.96%
Jeollanam-do	0.212	0.221	0.234	0.220	0.226	59.27%	44.13%	95.69%	0.00%	97.90%
South Korea	0.352	0.332	0.326	0.338	0.337	61.41%	28.73%	73.72%	0.00%	76.56%

**Table 8:** R-squared of machine learning models.

Machine Learning Models	R^2^
Linear Regression	0.791
SVR	0.803
BPNN	0.832
Decision Tree	0.837
Random Forest	0.848
GBDT	0.813
XGBoost	0.869
LightGBM	0.789
AdaBoost	0.855
Bagging	0.847
**Average Value**	0.828

**Table 9:** Prediction results of the low-carbon economic efficiency value in China from 2020 to 2024.

	2020	2021	2022	2023	2024
Guangdong	0.901	0.721	0.791	0.896	0.927
Jiangsu	0.901	0.835	0.783	0.921	0.978
Beijing	0.129	0.129	0.129	0.129	0.129
Shanghai	0.122	0.128	0.129	0.129	0.129
Fujian	0.093	0.094	0.105	0.115	0.115
Zhejiang	0.093	0.091	0.088	0.087	0.093
Hainan	0.080	0.080	0.080	0.080	0.080
Chongqing	0.085	0.082	0.080	0.080	0.080
Hubei	0.078	0.079	0.080	0.080	0.080
Shaanxi	0.078	0.079	0.079	0.080	0.080
Jiangxi	0.077	0.078	0.078	0.080	0.080
Hunan	0.077	0.077	0.077	0.078	0.080
Anhui	0.076	0.076	0.077	0.077	0.078
Yunnan	0.072	0.072	0.071	0.072	0.075
Sichuan	0.071	0.072	0.072	0.072	0.074
Henan	0.069	0.069	0.069	0.067	0.067
Shandong	0.069	0.069	0.071	0.070	0.073
Guizhou	0.067	0.064	0.062	0.061	0.059
Xinjiang	0.063	0.060	0.059	0.059	0.059
Tianjin	0.060	0.060	0.058	0.058	0.058
Guangxi	0.060	0.059	0.058	0.058	0.058
Qinghai	0.060	0.059	0.058	0.058	0.058
Gansu	0.059	0.058	0.058	0.058	0.058
Shanxi	0.059	0.058	0.058	0.058	0.058
Hebei	0.058	0.058	0.058	0.058	0.058
Inner Mongolia	0.059	0.058	0.058	0.058	0.058
Ningxia	0.058	0.058	0.058	0.058	0.058
Liaoning	0.058	0.058	0.058	0.058	0.058
Jilin	0.058	0.058	0.058	0.058	0.058
Heilongjiang	0.058	0.058	0.058	0.058	0.058
China	0.128	0.120	0.121	0.129	0.132

**Table 10:** Prediction results of the low-carbon economic efficiency value in Japan from 2020 to 2024.

	2020	2021	2022	2023	2024
Tokushima	0.887	0.909	0.976	0.911	0.907
Tottori	0.948	0.902	0.923	0.934	0.926
Tokyo	0.902	0.916	0.940	0.929	0.912
Yamanashi	0.922	0.949	0.937	0.910	0.916
Nara	0.924	0.929	0.931	0.932	0.930
Saga	0.859	0.915	0.975	0.933	0.916
Kochi	0.741	0.746	0.784	0.852	0.970
Shimane	0.707	0.744	0.801	0.795	0.928
Yamagata	0.688	0.710	0.728	0.742	0.796
Nagasaki	0.683	0.674	0.654	0.665	0.694
Fukui	0.606	0.609	0.609	0.608	0.600
Kyoto	0.612	0.606	0.606	0.600	0.606
Nagano	0.579	0.575	0.535	0.567	0.577
Shiga	0.600	0.593	0.596	0.608	0.603
Ishikawa	0.592	0.601	0.580	0.607	0.607
Gunma	0.602	0.598	0.601	0.610	0.598
Tochigi	0.601	0.581	0.602	0.606	0.602
Kagawa	0.555	0.538	0.584	0.569	0.564
Kagoshima	0.563	0.599	0.602	0.601	0.597
Okinawa	0.556	0.539	0.576	0.572	0.568
Toyama	0.541	0.552	0.587	0.568	0.572
Akita	0.521	0.487	0.490	0.502	0.495
Miyagi	0.460	0.478	0.471	0.464	0.467
Miyazaki	0.550	0.522	0.501	0.525	0.514
Kumamoto	0.499	0.484	0.474	0.481	0.472
Hokkaido	0.496	0.542	0.566	0.520	0.532
Wakayama	0.500	0.531	0.493	0.483	0.491
Shizuoka	0.488	0.472	0.478	0.477	0.469
Gifu	0.474	0.477	0.473	0.466	0.472
Fukushima	0.480	0.466	0.470	0.467	0.467
Osaka	0.477	0.473	0.476	0.474	0.470
Saitama	0.462	0.476	0.478	0.462	0.471
Iwate	0.486	0.463	0.476	0.483	0.463
Niigata	0.462	0.474	0.480	0.459	0.471
Aomori	0.434	0.451	0.450	0.460	0.458
Aichi	0.456	0.452	0.456	0.467	0.457
Fukuoka	0.433	0.453	0.447	0.463	0.458
Hyogo	0.466	0.453	0.463	0.464	0.469
Ibaraki	0.416	0.437	0.449	0.458	0.456
Mie	0.402	0.399	0.452	0.441	0.456
Kanagawa	0.414	0.435	0.425	0.435	0.429
Chiba	0.398	0.615	0.486	0.469	0.499
Ehime	0.372	0.389	0.360	0.375	0.364
Yamaguchi	0.367	0.369	0.336	0.362	0.365
Hiroshima	0.368	0.386	0.383	0.347	0.359
Oita	0.322	0.353	0.334	0.324	0.324
Okayama	0.336	0.324	0.324	0.322	0.321
Japan	0.558	0.567	0.571	0.570	0.576

**Table 11:** Prediction results of the low-carbon economic efficiency value in South Korea from 2020 to 2024.

	2020	2021	2022	2023	2024
Sejong	0.728	0.745	0.843	0.907	0.963
Jeju	0.468	0.455	0.466	0.497	0.473
Seoul	0.458	0.46	0.468	0.455	0.458
Gwangju	0.498	0.477	0.483	0.479	0.487
Daejeon	0.368	0.371	0.372	0.373	0.370
Daegu	0.325	0.320	0.308	0.302	0.303
Ulsan	0.305	0.303	0.297	0.287	0.301
Busan	0.298	0.295	0.296	0.296	0.296
Gyeongsangnam-do	0.283	0.283	0.282	0.283	0.295
Chungcheongbuk-do	0.280	0.282	0.282	0.282	0.282
Gyeonggi-do	0.275	0.274	0.273	0.273	0.273
Jeollabuk-do	0.279	0.28	0.281	0.281	0.283
Gyeongsangbuk-do	0.264	0.266	0.266	0.267	0.267
Gangwon-do	0.249	0.264	0.260	0.265	0.266
Incheon	0.252	0.242	0.243	0.243	0.243
Chungcheongnam-do	0.225	0.231	0.229	0.242	0.238
Jeollanam-do	0.222	0.224	0.227	0.214	0.210
South Korea	0.340	0.340	0.346	0.350	0.353

## Data Availability

The data of this study can be obtained by contacting the author, email address: zhangzhishuo@bfsu.edu.cn.

## Appendix A

**Table A1:** Machine Learning Model Parameter Optimization Results.

Machine Learning Model	Optimal Parameter Combination
Linear Regression	normalize = True
SVR	C = 19, gamma = 0.3
BPNN	hidden_layer_sizes = [5], max_iter = 100, solver = ‘lbfgs’
Decision Tree	max_depth = 8, max_features = 2
Random Forest	max_depth = 9, n_estimators = 500
GBDT	max_depth = 5, n_estimators = 500
XGBoost	learning_rate = 0.01, n_estimators = 500, max_depth = 5, subsample = 0.6
LightGBM	max_depth = 6, subsample = 0.6)
AdaBoost	learning_rate = 0.001, n_estimators = 100
Bagging	base_estimator = Decision Tree Regressor (max_depth = 7), n_estimators = 500
